# Opioid Receptors Gene Polymorphism and Heroin Dependence in Iran

**DOI:** 10.29252/nirp.bcn.9.2.101

**Published:** 2018

**Authors:** Sara Soleimani Asl, Amir Roointan, Hugo Bergen, Shayan Amiri, Parastoo Mardani, Niloufar Ashtari, Ronak Shabani, Mehdi Mehdizadeh

**Affiliations:** 1. Neurophysiology Research Center, Hamadan University of Medical Sciences, Hamadan, Iran.; 2. Department of Medical Biotechnology, School of Advanced Medical Sciences and Technologies, Shiraz University of Medical Sciences, Shiraz, Iran.; 3. Department of Human Anatomy and Cell Sciences, Rady Faculty of Health Sciences, University of Manitoba, Winnipeg, Manitoba, Canada.; 4. Department of Pharmacology, School of Medicine, Tehran University of Medical Sciences, Tehran, Iran.; 5. Department of Biology, Faculty of Science, Tehran Branch, Payame Noor University, Tehran, Iran.; 6. Cellular and Molecular Research Center, Department of Anatomy, Faculty of Advanced Technologies in Medicine, Iran University of Medical Sciences, Tehran, Iran.

**Keywords:** μ-opioid receptor, κ-opioid receptor, δ-opioid receptor, Single nucleotide polymorphism, Heroin

## Abstract

**Introduction:**

Genes often have multiple polymorphisms that interact with each other and the environment in different individuals. Variability in the opioid receptors can influence opiate withdrawal and dependence. In humans, A118G Single Nucleotide Polymorphisms (SNP) on μ-Opioid Receptor (MOR), 36 G>T in κ-Opioid Receptor (KOR), and T921C in the δ-Opioid Receptor (DOR) have been found to associate with substance dependence.

**Methods:**

To investigate the association between opioid receptors gene polymorphism and heroin addiction, 100 control subjects with no history of opioid use, and 100 heroin addicts (50% males and 50% females) in Tehran (capital of Iran), were evaluated. A118G, 36 G>T, and T921C SNPs on the MOR, KOR, DOR genes, respectively, were genotyped by sequencing.

**Results:**

We found no differences in either allele or genotype frequency for MOR, KOR and DOR genes SNPs between controls and subjects addicted to heroin.

**Conclusion:**

The relationships among polymorphisms may be important in determining the risk profile for complex diseases such as addiction, but opioid addiction is a multifactorial syndrome which is partially hereditary and partially affected by the environment.

## 1. Introduction

While severe social and economic problems will result from using illegal drugs in the society, pathogenesis of addictive disorders and mechanisms of therapeutic strategies for drug addicts have not been well understood yet (Mayer et al., 1997). Transition from an occasional user to an addict depends on the drug user, drug, and environment (Yuferov, Levran, Proudnikov, Nielsen, & Kreek, 2010). The μ-Opioid Receptor (MOR), κ-Opioid Receptor (KOR) and δ-Opioid Receptor (DOR) mediate the impact of opioids including tolerance, analgesia, dependence, and reward (Stein, 2016). Several lines of evidence indicate that genetic variations in the MOR gene (OPRM1), KOR gene (OPRK1) and DOR gene (OPRD1) can influence the expression, structure, or function of the receptors and ultimately result in increased or decreased susceptibility to opioid dependency (Crist, Doyle, Kampman, & Berrettini, 2016; Sharafshah et al., 2017).

Single nucleotide polymorphisms (SNPs) in OPRM1, OPRK1, and OPRD1 are candidates for their role in mediating differences in the opioid addiction. Genetic variation in these genes may create or function (which might result in receptors with altered expression) structured decrease or increase vulnerability to reliance on substance (Bond et al., 1998; Koch et al., 2000; Wang, Quillan, Winans, Lucas, & Sadée, 2001), and impact treatment response to opioid antagonists (Oslin et al., 2003). A number of studies have reported that A118G, in the first exon of the OPRM1 gene can be influential in individual susceptibility toward opioid dependency (Kreek, Bart, Lilly, Laforge, & Nielsen, 2005). Also, 36 G>T SNP at exon 1 of the KOR gene has been reported to contribute in predisposition to voluntary alcohol-drinking behavior in experimental animals (Saito et al., 2003).

In humans, this SNP has been found to be substantially associated with a population of heroin addicts of West European, Caucasian origin (Gerra et al., 2007). It has been reported that T921C SNP on the 3 exon OPRD1 is associated with increased substance abuse (Gelernter & Kranzler, 2000; Mayer et al., 1997). In addition, dependence on opioids is a medical and social problem in the world as well as Iran (Degenhardt et al., 2013; Majdzadeh et al., 2009; Shadnia, Soltaninejad, Heydari, Sasanian, & Abdollahi, 2008). Thus, further research is needed to recognize genetic variables that contribute to the progress of opioid addiction, to confirm probable genetic associations and to increase the neurobiological understanding of opioid dependence in order to find more potent analgesics with minimal unwanted actions. The principle objective of this research was to recognize genetic polymorphisms associated with the exclusive vulnerability toward opioid addiction and reproduce previous research in this field. Identifying these genetic markers will help detect people at risk for opiate addiction and provide better treatment options for them.

## 2. Methods

### 2.1. Study subjects

One hundred control subjects with no history of opioid use and one hundred heroin addicts (50% males and 50% females, aged 20–45 years) volunteered for the study in Tehran, Iran. The control group was recruited from university students and employees while the study group was recruited from drug rehabilitation centers in Tehran. Both groups participated in the research with their written consent. The participants were not paid and agreed to participate in the study voluntarily. In drug dependent individuals, heroin abuse was confirmed by psychiatric examination. Exclusion criteria included polydrug abusers, utilization of other narcotics, excessive alcohol in-take, and psychotropic factors.

### 2.2. DNA extraction and PCR amplification

Twenty milliliters of EDTA-treated blood was obtained from peripheral vein of subjects and control groups. We used a DNA kit (Cinnagen, Tehran, Iran) for DNA extraction according to the manufacturer’s protocols. Briefly, lysis buffer was added to the sample and vortexed. Then, the precipitation solution was added to the sample and the solution transferred to a collection tube and centrifuged at 13000 rpm. The spin column was washed and the DNA was eluted to a tube by elution buffer.

### 2.3. Selection and genotyping of SNPs

The SNPs A118G in exon 1 of the OPRM1 (Grösch, Niederberger, Lötsch, Skarke, & Geisslinger, 2001), 36 G>T in the exon 2 of the OPRK1 (Gerra et al., 2007), and T921C in the exon 1 of the OPRD1 (Franke et al., 1999) were amplified using primers provided in Table 1. PCR amplifications were performed in 20 μL PCR reaction system consisting of 20 mM Tris–HCl pH 8.0, 50 mM EDTA, 0.2 mM dNTPs, 1.5 mM MgCl2, 0.5 μmol each primer (forward and reverse) and 2.5 units of Taq polymerase (Kumar, Chakraborty, & Das, 2012). The PCR reactions were programmed as follows. For OPRD, we used an initial denaturation at 95ºC for 3 min, 40 cycles at 95ºC for 50 s, 66ºC for 90 s and 72ºC for 90 s. The reaction was terminated by an elongation period at 72ºC for 6 min. For OPRM, we used an initial denaturation at 95ºC for 5 min, followed by 45 cycles at 95ºC for 10 s, 50ºC for 150 s and 72ºC for 15 s with a final elongation time at 72ºC for 6 min. The same annealing temperature was used for β-actin. For OPRK, we used the same program as OPRM with 43 cycles and 55ºC annealing temperature. PCR products were separated by electrophoresis in 1.5% agarose gel at 100 V. PCR products size (301, 300, and 294 bp for OPRM, OPRK, and OPRD, respectively) confirmation was performed using a 1% agarose gel stained by ethidium-bromide and visualized by UV light. Automated DNA sequencing was performed using the primer described above and the Applied Biosystems TaqMan platform (Source Bioscience Sequencing, Cambridge, UK). Sequences were assembled by using Chromas.

**Table 1.** List of primers in the study

**Gene**	**SNP**	**Forward Primers**	**Reverse Primers**
OPRM	A118G	5′-GCTTGGAACCCGAAAAGTC-3′	5′-GTAGAGGGCCATGATCGTGAT-3
OPRK	G36T	5′-CGTGCGCTGAGAGGCGGGGG-3	5′-GCCGTGATGATGACCGGGATG-3
OPRD	T921C	5′-GGTGTGCATGCTCCAGTTCC-3	5′-CGCGCCGGTCGATGTCCACC-3

### 2.4. Statistical analysis

We used SPSS 20 (SPSS Inc., Chicago, IL, USA) to analyze the obtained data. The genotype frequencies of OPRM, OPRD, and OPRK markers were compared between cases and controls through the single-locus case– control test function in PowerMarker.

## 3. Results

**Figure 1.**
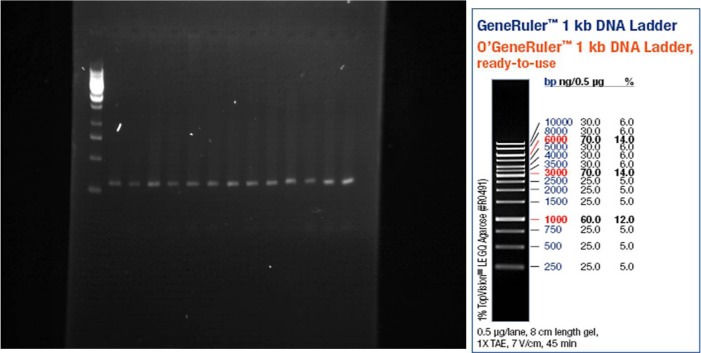
OPRM gene expression on agarose 1% gel (301 bp)

**Figure 2.**
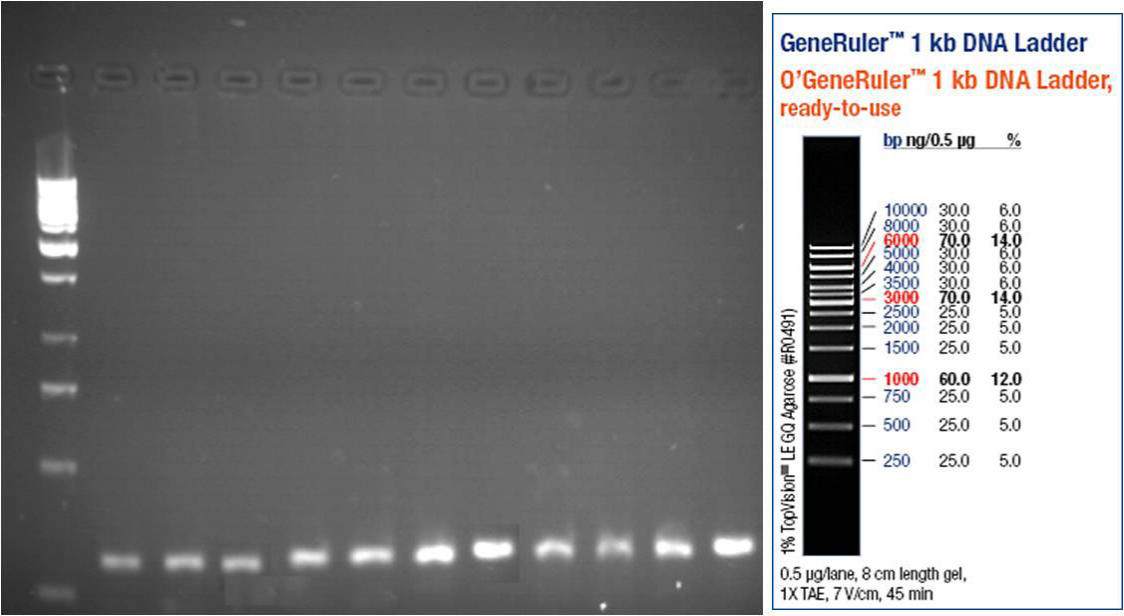
OPRK gene expression on agarose 1% gel (300 bp)

**Figure 3.**
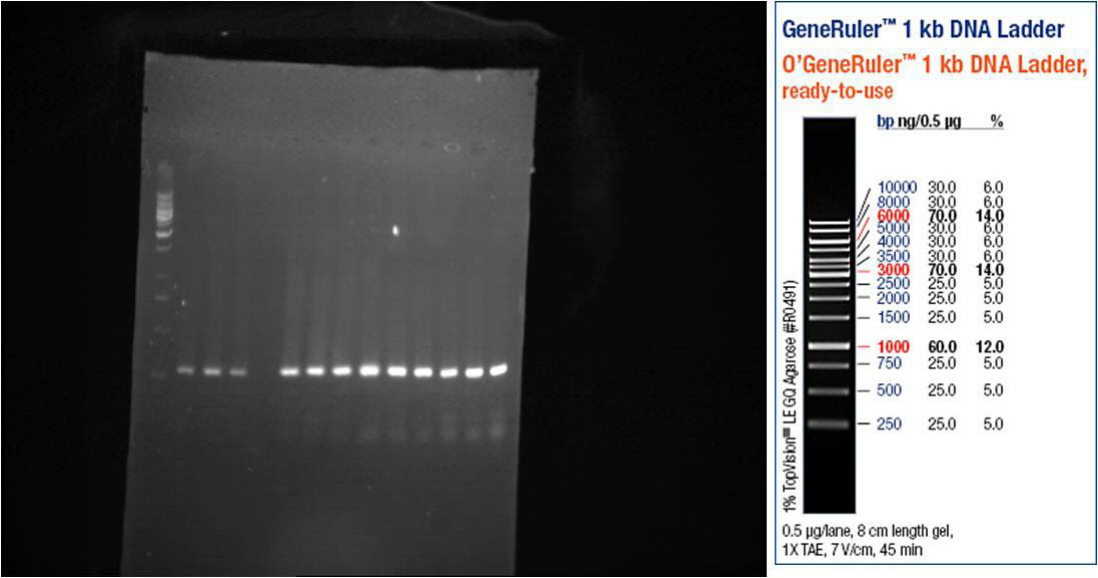
OPRD gene expression on agarose 1% gel (294 bp)

After separation of PCR products on 1% agarose gel, the bands were visualized by UV light. As shown in Figures 1, 2, and 3, the PCR products size were 301, 300, and 294 bp for OPRM, OPRK, and OPRD, respectively. The results of automated DNA sequencing were assembled by using Chromas (Table 2). Analysis of sequencing of OPRM showed no difference between the case and control group and the all of cases expressed the A nucleotide. Similar to OPRM, there was no variation in OPRK and OPRD sequencings. Finally, our results showed no association between heroin dependence and A118G, G36 T, and T921 C SNPs in the OPRM, OPRK, and OPRD, respectively. A possible explanation for these results may lie in the diversity of allele frequency across and within populations.

**Table 2.** Sequencing of the genes

**Gene**	**Sequencing**
OPRM	GCTTGGAACCCGAAAAGTCTCGGTGCTCCTGGCTACCTCGCACAGCGGTGCCCGCCCGGCCGTCAGTACCATGGACAGCAGCGCT-GCCCCCACGAACGCCAGCAATTGCACTGATGCCTTGGCGTACTCAAGTTGCTCCCCAGCACCCAGCCCCGGTTCCTGGGTCAACTT-GTCCCACTTAGATGGCAACCTGTCCGACCCATGCGGTCCGAACCGCACCGACCTGGGCGGGAGAGACAGCCTGTGCCCTCCGACCG-GCAGTCCCTCCATGATCACGGCCATCACGATCATGGCCCTCTAC
OPRK	AGTGGGAGACGTGCGCTGAGAGGCGGGGGCTGCGCTCGGCGGAACAGCAGCCCTCGGGCGGAGAGCGGGGCCGGGGTCCGAGA-GCAGGTGATGCCAAGAGCTGAGCGGGACTCGTGAGCGCGCGGTTCAGCACCTACCAGGGCGTCCCGTAAAAAACCTCGCCTTCGCCT-GTCTCTGGGAACCATAGGTAAGCTTTGGGCTTTCGAGGTGCAGTTCTAGGTAGAGCTCCGTGCTGGGAGGTGGGAAGGGGGCTT-GACCCTGGGGACTCAGGCAGTCTGGG
OPRD	GGTGTGCATGCTCCAGTTCCCCAGCCCCAGCTGGTACTGGGACACGGTGACCAAGATCTGCGTGTTCCTCTTCGCCTTCGTGGTGCCCATCCT-CATCATCACCGTGTGCTATGGCCTCATGCTGCTGCGCCTGCGCAGTGTGCGCCTGCTGTCGGGCTCCAAGGAGAAGGACCGCAGCCTGCG-GCGCATCACGCGCATGGTGCTGGTGGTTGTGGGCGCCTTCGTGGTGTGTTGGGCGCCCATCCACATCTTCGTCATCGTCTGGACGCTGGTG-GACATCGACCGGCGCG

## 4. Discussion

Addiction to drugs is influenced by physiological, psychological, pharmacological, genetic and environmental factors. Furthermore, genetic parameters play an important role in the pathogenesis of opioid addiction; heritability nature of opioid abuse and or addiction ranges from 43% to 60% (Goldman, Oroszi, & Ducci, 2005; Li & Burmeister, 2009). Numerous lines of evidence has demonstrated a connection between opioid receptor variability and substance addiction in humans (Kowarik et al., 2012; Soto & Raingo, 2012). In this regard, Mayer et al. reported an association between DOR polymorphism and heroin dependence in men (Mayer et al., 1997). They showed that allele C was more frequent in German Caucasian heroin addicts than in controls. Studies on experimental animals have shown that genetic polymorphisms in KORs play a role in having tendency toward voluntary alcohol-drinking behavior (Saito et al., 2003; Vadasz, Saito, Gyetvai, & Mikics, 2000). In addition, preliminary studies in humans revealed that 36 G>T SNP on the KOR gene (hOPRK1) displays a strong association with A variety of addictive diseases (Yuferov et al., 2004). According to another study, heroin dependency is shown to be correlated with a high frequency of allele 36 G>T SNP in the exon 2 of the human KOR gene (hOPRK1), proposing that the T allele might predispose people to addictive behavior or to personality traits at risk for substance abuse (Gerra et al., 2007).

The present findings indicate no association between opioid dependency and novel polymorphisms A118G, 36 G>T, 921C SNP in MOR, KOR, DOR genes, respectively in contrast to the previously mentioned study. There are number of studies that consistent with our results. In this context, Bond and colleagues found no significant differences in A118G allele frequency between opioid dependent and non-dependent subjects with all ethnic groups combined (Bond et al., 1998). In another study on a European population, no significant association was shown between this SNP and opioid addiction (Beer et al., 2013). However in subgroup analysis by ethnicity, the A118G minor allele (G) frequency was significantly higher in non-opioid dependent Hispanic subjects (Bond et al., 1998). A study by Bart et al. also corroborated this association on a Swedish population of opiate-dependent and control subjects, demonstrating higher frequencies of the minor allele in opiate-dependent users (Bart et al., 2004).

Study on MOR and KOR genes in Taiwanese population revealed no significant differences in genotype frequency or allele on subjects with alcohol dependency and controls (Loh, Fann, Chang, Chang, & Cheng, 2004). In addition, Franke et al. findings do not support the hypothesis that the C allele elevates the risk of substance abuse, neither for alcohol dependence nor for heroin dependence (Franke et al., 1999). In a European American population study by Zhang et al., no significant association was shown between this SNP with alcohol, cocaine, and opioid dependence (Zhang, Kranzler, Yang, Luo, & Gelernter, 2007). In contrast to these reports, Mayer et al. showed that the abundance a silent T to C substitution in site 921 of the δ-opioid receptor gene was significantly higher in heroin addicts than in control population (Mayer et al., 1997). The stressful events, personality disorders and negative emotional relationships, such as parent-child relationships, are considered predictors of addictive behaviors (Conner, Hellemann, Ritchie, & Noble, 2010). This is a good evidence for complex diseases where the genetic etiology is indicative of multifactorial syndrome. The genetic effects on any behavioral outcome are influenced by exposure of individual to a certain environment. Thus, when interpreting the findings, interactions between environmental and genetic factors should be considered, for example, the tendency of individuals to purposefully adapt to specific environments.

Our findings suggest that opioid receptor gene polymorphisms do not have a significant influence on the progress of heroin dependence among the Iranian population. Further studies are necessary to examine variables of opioid receptors genotype and its relation with individuals’ characteristics at risk to addictive behavior.
